# *O*-Pivaloyl diphenyl­seleno­phosphinate

**DOI:** 10.1107/S1600536809009295

**Published:** 2009-03-25

**Authors:** Grzegorz Cholewinski, Jaroslaw Chojnacki, Jerzy Pikies, Janusz Rachon

**Affiliations:** a Chemical Faculty, Gdansk University of Technology, Narutowicza 11/12, Gdansk PL-80233, Poland

## Abstract

The title compound, C_17_H_19_O_2_PSe, was obtained in the reaction of the diphenyl­monoseleno­phosphinic acid ammonium salt with pivaloyl chloride. The P—Se bond length of 2.0769 (11) Å is normal, while the P—O bond length of 1.650 (3) Å is longer than in related *O*-alkyl and *O*-aryl derivatives. One phenyl ring is periplanar to the Se—P—C plane, while the dihedral angle between the two phenyl rings is *ca* 73°. The carbonyl group is in a synperiplanar position [torsion angle = 8.9 (6)°] to one of the methyl groups of the pivaloyl group. This is the first *O*-acyl derivative of diphenyl­monoseleno­phosphinic acid characterized by X-ray structural analysis.

## Related literature

Syntheses and the chemical properties of *O*-acyl monoseleno­phosphates have already been described by Rachon *et al.* (2005 ▶). For other monoseleno­phosphates, such as *O*-alkyl or *O*-aryl esters, see: Lepicard *et al.* (1969 ▶); Balakrishna *et al.* (2002 ▶, 2005 ▶); Mague *et al.* (2007 ▶). For details of the Cambridge Crystallographic Database, see: Allen (2002 ▶).
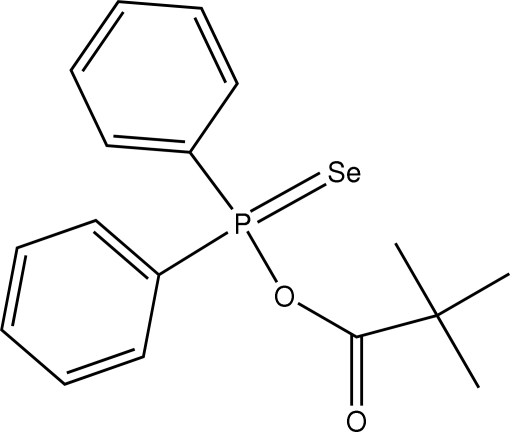


## Experimental

### 

#### Crystal data

C_17_H_19_O_2_PSe
*M* *_r_* = 365.25
Monoclinic, 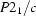

*a* = 9.6212 (5) Å
*b* = 10.3914 (5) Å
*c* = 17.1087 (9) Å
β = 99.618 (5)°
*V* = 1686.45 (15) Å^3^
*Z* = 4
Mo *K*α radiation
μ = 2.32 mm^−1^
*T* = 120 K
0.22 × 0.2 × 0.12 mm

#### Data collection

Oxford Diffraction KM-4-CCD diffractometer
Absorption correction: multi-scan (*CrysAlis RED*; Oxford Diffraction, 2008 ▶) *T*_min_ = 0.588, *T*_max_ = 0.760
12450 measured reflections
3674 independent reflections
2596 reflections with *I* > 2σ(*I*)
*R*_int_ = 0.06

#### Refinement

*R*[*F* ^2^ > 2σ(*F* ^2^)] = 0.057
*wR*(*F* ^2^) = 0.147
*S* = 0.97
3674 reflections
193 parameters
H-atom parameters constrained
Δρ_max_ = 2.18 e Å^−3^
Δρ_min_ = −0.65 e Å^−3^

### 

Data collection: *CrysAlis CCD* (Oxford Diffraction 2008 ▶); cell refinement: *CrysAlis RED* (Oxford Diffraction 2008 ▶); data reduction: *CrysAlis RED*; program(s) used to solve structure: *SHELXS97* (Sheldrick, 2008 ▶); program(s) used to refine structure: *SHELXL97* (Sheldrick, 2008 ▶); molecular graphics: *ORTEP-3 for Windows* (Farrugia, 1997 ▶); software used to prepare material for publication: *WinGX* (Farrugia, 1999 ▶) and *PLATON* (Spek, 2009 ▶).

## Supplementary Material

Crystal structure: contains datablocks global, I. DOI: 10.1107/S1600536809009295/ez2163sup1.cif

Structure factors: contains datablocks I. DOI: 10.1107/S1600536809009295/ez2163Isup2.hkl

Additional supplementary materials:  crystallographic information; 3D view; checkCIF report

## Figures and Tables

**Table 1:** Comparison of the geometry of the title compound with related compounds (Å, °)

CSD refcode(Allen, 2002 ▶)	P—Se	P—O	Ph–Ph dihedral	Smaller torsion	Reference
	2.0769 (11)	1.650 (3)	72.64 (14)	7.0 (4)	This work
MPSEPO	2.080	1.619	82.62	4.15	Lepicard *et al.* (1969 ▶)
MUMFUV	2.072	1.624	80.93	13.32	Balakrishna *et al.* (2002 ▶)
	2.070	1.612	75.01	22.34	
RAMXEJ	2.089	1.596	78.65	8.84	Balakrishna *et al.* (2005 ▶)
	2.079	1.585	78.49	6.58	
YIQOM	2.089	1.620	70.15	6.15	Mague *et al.* (2007 ▶)

**Fig. 1.**
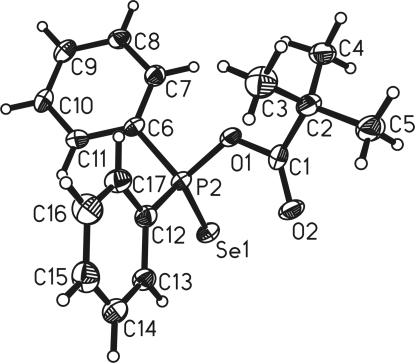
View of the title compound showing the atom-numbering scheme (50% probability displacement ellipsoids).

## supplementary crystallographic information

### Comment

*O*-acyl monoselenophosphates were studied in a search for potential selenoacylating agents. *O*-pivaloyl-diphenylmonoselenophosphinate, C_17_H_19_O_2_PSe, was obtained in the reaction of diphenylmonoselenophosphinic acid ammonium salt with pivaloyl chloride (Rachon *et al.*, 2005). The P—Se bond length is normal for a double bond, while the P—O bond is rather long when compared with the related *O*-alkyl and *O*-aryl derivatives (see Table 1). One phenyl ring is placed periplanar to the Se—P—C plane, while the dihedral angle between the two phenyl rings is relatively small. The carbonyl group is in a synperiplanar position [torsion angle = 8.9 (6)°] to one of methyl groups in the pivaloyl group.

This compound, together with *O-p*-chlorobenzoyl-diphenylselenophosphinate, reported in the following paper, are the first structures determined by X-ray diffraction of *O*-acyl derivatives of diphenylmonoselenophosphinic acid reported. Only four related *O*-alkyl and *O*-aryl derivatives were characterized by *x*-ray study so far: methyl diphenylselenophosphinate (Lepicard *et al.*, 1969), 1,4-bis(diphenyl(seleno)phosphinito)cyclohexane (Balakrishna *et al.*, 2005); 1,1'-methylene-bis(2-((diphenylphosphoroselenoyl)oxy)naphthalene) (Balakrishna *et al.*, 2002) and *O*-2-naphthyl diphenylselenophosphinate (Mague *et al.*, 2007).

### Experimental

*O*-Pivaloyl diphenylmonoselenophosphinate was obtained in the reaction of diphenylmonoselenophosphinic acid ammonium salt with pivaloyl chloride with 43% yield (Rachon *et al.*, 2005, compound numbered as 2u, melting point 63-65 °C). Relevant ^1^H, ^13^C, ^31^P NMR, MS and IR spectra were recorded and are consistent with the formula anticipated - see the supporting information for the article cited.

### Refinement

Hydrogen atoms were placed in calculated positions and refined using a standard riding model. C–H bond lengths were set to 0.98 or 0.95 Å and *U**_iso_*(H) were set to 1.5 or 1.2 *U**_eq_*(C) for methyl or aromatic C–H groups, respectively.

The residual electron density peak is 0.84 Å from Se1, the deepest electron density hole is 1.24 Å from Se1.

### Crystal data

**Table:**

C_17_H_19_O_2_PSe	*F*(000) = 744
*M**_r_* = 365.25	*D*_x_ = 1.439 Mg m^−^^3^
Monoclinic, *P*2_1_/*c*	Melting point: 337(2) K
Hall symbol: -P 2ybc	Mo *K*α radiation, λ = 0.71073 Å
*a* = 9.6212 (5) Å	Cell parameters from 5361 reflections
*b* = 10.3914 (5) Å	θ = 2.0–32.4°
*c* = 17.1087 (9) Å	µ = 2.32 mm^−^^1^
β = 99.618 (5)°	*T* = 120 K
*V* = 1686.45 (15) Å^3^	Fragment, colourless
*Z* = 4	0.22 × 0.2 × 0.12 mm

### Data collection

**Table:**

Oxford Diffraction KM-4-CCD diffractometer	3674 independent reflections
Radiation source: Mo Kα radiation	2596 reflections with *I* > 2σ(*I*)
graphite	*R*_int_ = 0.06
ω scans (0.75° width)	θ_max_ = 27°, θ_min_ = 2.3°
Absorption correction: multi-scan (*CrysAlis RED*; Oxford Diffraction, 2008)	*h* = −12→12
*T*_min_ = 0.588, *T*_max_ = 0.760	*k* = −12→13
12450 measured reflections	*l* = −21→19

### Refinement

**Table:**

Refinement on *F*^2^	Primary atom site location: structure-invariant direct methods
Least-squares matrix: full	Secondary atom site location: difference Fourier map
*R*[*F*^2^ > 2σ(*F*^2^)] = 0.057	Hydrogen site location: inferred from neighbouring sites
*wR*(*F*^2^) = 0.147	H-atom parameters constrained
*S* = 0.97	*w* = 1/[σ^2^(*F*_o_^2^) + (0.0979*P*)^2^] where *P* = (*F*_o_^2^ + 2*F*_c_^2^)/3
3674 reflections	(Δ/σ)_max_ = 0.001
193 parameters	Δρ_max_ = 2.18 e Å^−^^3^
0 restraints	Δρ_min_ = −0.65 e Å^−^^3^

### Special details

**Table:**

Geometry. All e.s.d.'s (except the e.s.d. in the dihedral angle between two l.s. planes) are estimated using the full covariance matrix. The cell e.s.d.'s are taken into account individually in the estimation of e.s.d.'s in distances, angles and torsion angles; correlations between e.s.d.'s in cell parameters are only used when they are defined by crystal symmetry. An approximate (isotropic) treatment of cell e.s.d.'s is used for estimating e.s.d.'s involving l.s. planes.

### Fractional atomic coordinates and isotropic or equivalent isotropic displacement parameters (Å^2^)

**Table:**

	*x*	*y*	*z*	*U*_iso_*/*U*_eq_	
P2	0.85844 (10)	0.75002 (9)	0.28847 (6)	0.0208 (2)	
Se1	0.78275 (4)	0.76292 (4)	0.16772 (2)	0.02698 (17)	
O1	0.8422 (2)	0.6100 (2)	0.33157 (15)	0.0243 (6)	
O2	0.6042 (3)	0.6037 (3)	0.30807 (16)	0.0310 (6)	
C1	0.7130 (4)	0.5607 (4)	0.3426 (2)	0.0243 (8)	
C2	0.7308 (4)	0.4557 (4)	0.4043 (2)	0.0274 (8)	
C3	0.7855 (5)	0.5221 (5)	0.4839 (2)	0.0424 (11)	
H3A	0.8789	0.5588	0.4826	0.064*	
H3B	0.7923	0.4588	0.5269	0.064*	
H3C	0.7203	0.5908	0.493	0.064*	
C4	0.8353 (4)	0.3542 (4)	0.3869 (3)	0.0341 (10)	
H4A	0.7989	0.3118	0.3365	0.051*	
H4B	0.8486	0.2903	0.4296	0.051*	
H4C	0.9258	0.3953	0.3835	0.051*	
C5	0.5874 (4)	0.3949 (4)	0.4078 (3)	0.0412 (11)	
H5A	0.5226	0.4609	0.4212	0.062*	
H5B	0.5984	0.3272	0.4482	0.062*	
H5C	0.5493	0.3574	0.356	0.062*	
C6	1.0474 (4)	0.7623 (3)	0.3126 (2)	0.0211 (8)	
C7	1.1309 (4)	0.6520 (4)	0.3187 (2)	0.0263 (8)	
H7	1.0889	0.569	0.3136	0.032*	
C8	1.2769 (4)	0.6654 (4)	0.3324 (2)	0.0273 (9)	
H8	1.3348	0.5908	0.3365	0.033*	
C9	1.3386 (4)	0.7856 (4)	0.3399 (2)	0.0298 (9)	
H9	1.4384	0.7935	0.3494	0.036*	
C10	1.2550 (4)	0.8946 (4)	0.3337 (2)	0.0277 (9)	
H10	1.2976	0.9773	0.3387	0.033*	
C11	1.1095 (4)	0.8839 (4)	0.3203 (2)	0.0242 (8)	
H11	1.0524	0.9589	0.3164	0.029*	
C12	0.7863 (4)	0.8624 (4)	0.3506 (2)	0.0254 (8)	
C13	0.6748 (4)	0.9438 (4)	0.3208 (2)	0.0262 (8)	
H13	0.6364	0.9409	0.266	0.031*	
C14	0.6194 (4)	1.0279 (4)	0.3691 (3)	0.0338 (10)	
H14	0.5447	1.084	0.3476	0.041*	
C15	0.6734 (4)	1.0307 (4)	0.4499 (3)	0.0357 (10)	
H15	0.6343	1.0875	0.4839	0.043*	
C16	0.7843 (5)	0.9505 (4)	0.4806 (3)	0.0376 (10)	
H16	0.8216	0.953	0.5356	0.045*	
C17	0.8406 (4)	0.8673 (4)	0.4316 (2)	0.0307 (9)	
H17	0.9169	0.8128	0.453	0.037*	

### Atomic displacement parameters (Å^2^)

**Table:**

	*U*^11^	*U*^22^	*U*^33^	*U*^12^	*U*^13^	*U*^23^
P2	0.0152 (5)	0.0257 (5)	0.0208 (5)	0.0002 (4)	0.0014 (4)	0.0001 (4)
Se1	0.0227 (2)	0.0355 (3)	0.0210 (2)	0.00040 (17)	−0.00147 (16)	0.00093 (15)
O1	0.0153 (13)	0.0272 (13)	0.0289 (14)	−0.0003 (10)	−0.0002 (10)	0.0038 (11)
O2	0.0159 (14)	0.0380 (16)	0.0371 (16)	0.0016 (12)	−0.0016 (12)	0.0088 (13)
C1	0.0201 (19)	0.0271 (19)	0.026 (2)	−0.0059 (16)	0.0044 (16)	−0.0059 (15)
C2	0.0211 (19)	0.035 (2)	0.025 (2)	−0.0008 (17)	0.0007 (16)	0.0055 (16)
C3	0.050 (3)	0.050 (3)	0.025 (2)	−0.004 (2)	0.003 (2)	0.0038 (19)
C4	0.026 (2)	0.035 (2)	0.040 (2)	0.0042 (18)	0.0028 (18)	0.0097 (18)
C5	0.023 (2)	0.046 (3)	0.054 (3)	−0.0007 (19)	0.006 (2)	0.017 (2)
C6	0.0152 (17)	0.0296 (19)	0.0183 (18)	0.0002 (14)	0.0024 (14)	0.0004 (14)
C7	0.023 (2)	0.0282 (19)	0.027 (2)	−0.0011 (16)	0.0032 (16)	0.0007 (15)
C8	0.020 (2)	0.031 (2)	0.031 (2)	0.0051 (16)	0.0040 (16)	−0.0002 (16)
C9	0.019 (2)	0.041 (2)	0.028 (2)	−0.0034 (17)	0.0029 (16)	−0.0027 (18)
C10	0.023 (2)	0.033 (2)	0.025 (2)	−0.0076 (16)	0.0008 (16)	−0.0022 (16)
C11	0.022 (2)	0.0279 (19)	0.0225 (19)	0.0044 (16)	0.0031 (15)	0.0010 (15)
C12	0.020 (2)	0.0263 (19)	0.030 (2)	0.0003 (16)	0.0039 (16)	−0.0025 (16)
C13	0.0209 (19)	0.028 (2)	0.030 (2)	0.0002 (16)	0.0040 (16)	0.0016 (16)
C14	0.021 (2)	0.032 (2)	0.048 (3)	−0.0008 (17)	0.0052 (19)	−0.0006 (19)
C15	0.035 (2)	0.035 (2)	0.039 (3)	−0.0017 (19)	0.013 (2)	−0.0078 (19)
C16	0.047 (3)	0.039 (2)	0.028 (2)	0.002 (2)	0.0062 (19)	−0.0013 (18)
C17	0.030 (2)	0.034 (2)	0.026 (2)	0.0029 (18)	0.0008 (17)	0.0022 (17)

### Geometric parameters (Å, °)

**Table:**

P2—O1	1.650 (3)	C7—C8	1.392 (5)
P2—C12	1.795 (4)	C7—H7	0.95
P2—C6	1.801 (4)	C8—C9	1.381 (6)
P2—Se1	2.0769 (11)	C8—H8	0.95
O1—C1	1.386 (4)	C9—C10	1.383 (6)
O2—C1	1.200 (4)	C9—H9	0.95
C1—C2	1.507 (5)	C10—C11	1.384 (5)
C2—C4	1.521 (5)	C10—H10	0.95
C2—C5	1.527 (5)	C11—H11	0.95
C2—C3	1.539 (5)	C12—C13	1.394 (5)
C3—H3A	0.98	C12—C17	1.398 (5)
C3—H3B	0.98	C13—C14	1.370 (5)
C3—H3C	0.98	C13—H13	0.95
C4—H4A	0.98	C14—C15	1.394 (6)
C4—H4B	0.98	C14—H14	0.95
C4—H4C	0.98	C15—C16	1.386 (6)
C5—H5A	0.98	C15—H15	0.95
C5—H5B	0.98	C16—C17	1.377 (6)
C5—H5C	0.98	C16—H16	0.95
C6—C7	1.393 (5)	C17—H17	0.95
C6—C11	1.395 (5)		
			
O1—P2—C12	103.55 (16)	C7—C6—P2	120.4 (3)
O1—P2—C6	97.32 (15)	C11—C6—P2	119.1 (3)
C12—P2—C6	107.07 (17)	C8—C7—C6	118.9 (3)
O1—P2—Se1	117.24 (10)	C8—C7—H7	120.5
C12—P2—Se1	116.17 (13)	C6—C7—H7	120.5
C6—P2—Se1	113.35 (13)	C9—C8—C7	120.8 (4)
C1—O1—P2	122.8 (2)	C9—C8—H8	119.6
O2—C1—O1	121.6 (3)	C7—C8—H8	119.6
O2—C1—C2	127.0 (3)	C8—C9—C10	119.9 (4)
O1—C1—C2	111.3 (3)	C8—C9—H9	120
C1—C2—C4	111.5 (3)	C10—C9—H9	120
C1—C2—C5	109.3 (3)	C9—C10—C11	120.4 (4)
C4—C2—C5	110.6 (3)	C9—C10—H10	119.8
C1—C2—C3	106.1 (3)	C11—C10—H10	119.8
C4—C2—C3	110.1 (3)	C10—C11—C6	119.6 (3)
C5—C2—C3	109.2 (3)	C10—C11—H11	120.2
C2—C3—H3A	109.5	C6—C11—H11	120.2
C2—C3—H3B	109.5	C13—C12—C17	118.5 (4)
H3A—C3—H3B	109.5	C13—C12—P2	121.9 (3)
C2—C3—H3C	109.5	C17—C12—P2	119.5 (3)
H3A—C3—H3C	109.5	C14—C13—C12	121.4 (4)
H3B—C3—H3C	109.5	C14—C13—H13	119.3
C2—C4—H4A	109.5	C12—C13—H13	119.3
C2—C4—H4B	109.5	C13—C14—C15	119.5 (4)
H4A—C4—H4B	109.5	C13—C14—H14	120.2
C2—C4—H4C	109.5	C15—C14—H14	120.2
H4A—C4—H4C	109.5	C16—C15—C14	119.9 (4)
H4B—C4—H4C	109.5	C16—C15—H15	120
C2—C5—H5A	109.5	C14—C15—H15	120
C2—C5—H5B	109.5	C17—C16—C15	120.2 (4)
H5A—C5—H5B	109.5	C17—C16—H16	119.9
C2—C5—H5C	109.5	C15—C16—H16	119.9
H5A—C5—H5C	109.5	C16—C17—C12	120.4 (4)
H5B—C5—H5C	109.5	C16—C17—H17	119.8
C7—C6—C11	120.4 (3)	C12—C17—H17	119.8
			
Se1—P2—O1—C1	−69.5 (3)	O2—C1—C2—C3	−108.5 (5)
C6—P2—O1—C1	169.4 (3)	O2—C1—C2—C4	131.6 (4)
C12—P2—O1—C1	59.8 (3)	O2—C1—C2—C5	8.9 (6)
Se1—P2—C6—C7	−92.1 (3)	P2—C6—C7—C8	175.9 (3)
Se1—P2—C6—C11	84.0 (3)	C11—C6—C7—C8	−0.1 (5)
O1—P2—C6—C7	31.8 (3)	P2—C6—C11—C10	−175.8 (3)
O1—P2—C6—C11	−152.1 (3)	C7—C6—C11—C10	0.3 (5)
C12—P2—C6—C7	138.5 (3)	C6—C7—C8—C9	0.0 (5)
C12—P2—C6—C11	−45.4 (3)	C7—C8—C9—C10	−0.1 (5)
Se1—P2—C12—C13	7.0 (4)	C8—C9—C10—C11	0.3 (5)
Se1—P2—C12—C17	−174.5 (3)	C9—C10—C11—C6	−0.4 (5)
O1—P2—C12—C13	−123.0 (3)	P2—C12—C13—C14	179.1 (3)
O1—P2—C12—C17	55.5 (4)	C17—C12—C13—C14	0.5 (6)
C6—P2—C12—C13	134.8 (3)	P2—C12—C17—C16	−178.3 (3)
C6—P2—C12—C17	−46.7 (4)	C13—C12—C17—C16	0.3 (6)
P2—O1—C1—O2	15.0 (5)	C12—C13—C14—C15	−1.3 (6)
P2—O1—C1—C2	−162.2 (2)	C13—C14—C15—C16	1.3 (6)
O1—C1—C2—C3	68.6 (4)	C14—C15—C16—C17	−0.5 (6)
O1—C1—C2—C4	−51.4 (4)	C15—C16—C17—C12	−0.3 (7)
O1—C1—C2—C5	−174.0 (3)		

### Table 1 Comparison of the geometry of the title compound with related compounds (Å, °)

**Table:**

CSD refcode	P—Se	P—O	Ph–Ph dihedral	Smaller torsion	Reference
(Allen, 2002)			Ph–Ph dihedral	Smaller torsion	
	2.0769 (11)	1.650 (3)	72.64 (14)	7.0 (4)	This work
MPSEPO	2.080	1.619	82.62	4.15	Lepicard *et al.* (1969)
MUMFUV	2.072	1.624	80.93	13.32	Balakrishna *et al.* (2002)
	2.070	1.612	75.01	22.34	
RAMXEJ	2.089	1.596	78.65	8.84	Balakrishna *et al.* (2005)
	2.079	1.585	78.49	6.58	
YIQOM	2.089	1.620	70.15	6.15	Mague *et al.* (2007)
